# Development and Validation of an Ion-Pair Reverse-Phase High-Performance Liquid Chromatography–Electrospray Ionization Mass Spectrometry Method for Determination of Purity of Nusinersen for Quality Control of Drug Substance or Drug Product

**DOI:** 10.3390/ijms27073301

**Published:** 2026-04-05

**Authors:** Mikhail Samoilov, Ekaterina Zubareva, Maksim Degterev

**Affiliations:** Generium JSC, 14, Vladimirskaya Street, 601125 Volginskiy, Russia; m.samoilov@generium.ru (M.S.); zubareva@ibcgenerium.ru (E.Z.)

**Keywords:** oligonucleotide, purity, mass spectrometry, Nusinersen, validation, electrospray ionization, QTOF, time-of-flight mass spectrometry, ion-pair reverse-phase high-performance liquid chromatography

## Abstract

In this study, an ion-pair reverse-phase high-performance liquid chromatography–electrospray ionization mass spectrometry (RP-HPLC-ESI-MS) method was optimized and validated for purity determination for the quality control of the proposed generic nusinersen oligonucleotide drug substance and drug product. The optimization and considerations of sample preparation, chromatographic and mass spectrometry conditions are discussed. The limit of detection was 2.5 × 10^−5^ mg/mL and the limit of quantitation was 4.9 × 10^−5^ mg/mL. The linearity of the signal (XIC) for all impurities was linear with correlation coefficients of R^2^ ≥ 0.9669. This study, associated with the development of therapeutic oligonucleotides, examines the subject of product-related impurities. The authors consider an ion-pair reverse-phase high-performance liquid chromatography in combination with mass spectrometry for impurity quantitative control. This study contributes to the field by elucidating several critical aspects that, while previously unaddressed in the existing literature, are essential for developing effective analytical methods.

## 1. Introduction

Synthetic oligonucleotides have emerged as promising candidates for several orphan diseases. Nucleic acid-based drugs combine high specificity and efficacy and low toxicity. More than twenty oligonucleotide therapeutics have now received regulatory approval worldwide [1].

Nusinersen is an antisense oligonucleotide that is used for the treatment of spinal muscular atrophy. It is a neuromuscular disease which progresses over time and is inherited in an autosomal recessive pattern. The disease is caused by mutations in the *SMN1* gene, which codes for the survival motor neuron protein. A reduction in the level of the protein causes disorders in neuromuscular synapses and axonal defects in motor neurons. These defects are manifested as progressive muscle atrophy, flaccid paresis, and complications in the musculoskeletal, respiratory, and digestive systems [2]. Nusinersen is a pathogenetic treatment medication that raises the level of the motor neuron survival protein, halting the disease’s progression and assisting patients in stabilizing and/or enhancing their motor function. In addition to having a well-researched positive safety profile, nusinersen has proven to be efficacious across a wide range of clinical trial programs [3,4,5].

The development of methods for impurity assessment is essential for quality control and patient safety for any medicinal product. In the case of synthetic oligonucleotides, this task is particularly challenging because of their relatively high molecular weight (typically 6–18 kDa) and the presence of numerous low-level product-related impurities, such as shortmers/longmers and degradation products, whose physicochemical properties are often very similar to those of the parent molecule. As noted by the FDA (Food and Drug Administration) and EMA (European Medicines Agency), such impurities frequently co-elute with the main peak and cannot always be fully resolved by conventional chromatographic methods; therefore, orthogonal analytical approaches, including LC-MS (liquid chromatography–mass spectrometry), are often required for their characterization and control [6,7,8]. Importantly, even minor structural changes in oligonucleotides may alter hybridization behavior, protein binding, distribution, and toxicity, making impurity control directly relevant to patient safety [9,10]. Unmodified oligonucleotides have short half-life clearance due to enzymatic (deoxyribonucleases or ribonucleases) and chemical (oxidation, hydrolysis, and deamidation) degradation. Chemical modification is carried out to improve stability, to minimize proinflammatory characteristics, or to boost binding affinity [11]. For example, phosphorothioate modifications ensure protection from nuclease degradation. Sugar modification at the 2′-position of the ring increases synthetic oligonucleotide stability. However, these modifications add additional complexity to the analysis of synthetic oligonucleotides.

The structure of oligonucleotide impurities is also complicated and variable. They might originate from raw materials or occur during the manufacturing and storage of the pharmaceutical substance or commercialized drug. Oligonucleotides with missing or extra nucleotides are commonly reported as impurities (*n* + 1, *n* − 1, and *n* − 2) [12,13]. Frequently, synthetic oligonucleotides have phosphorothioate linkages, which can undergo oxidation to the typical impurity, phosphate diester (P=S to P=O) [14]. Typical modifications of synthetic oligonucleotides also include nucleobase changes (loss of adenine or guanine), thymine replacement with CNET (N3-(2-cyanoethyl)thymine), and 2′-modification of the sugar moiety [15,16,17]. Several analytical methods have been published to identify and measure oligonucleotide impurities. To separate longmers and shortmers, for instance, several types of electrophoresis are available. Phosphodiester (P=O) impurities in phosphorothioate oligonucleotides can be measured using strong anion exchange chromatography [13]. However, electrophoresis and chromatography methods are inherently insufficiently selective. Complete resolution of each impurity is necessary for identification, but complete resolution is unattainable due to the quantity of impurities and their chemical similarity. To maximize the analytical resolution and selectivity, the primary approach for evaluating synthetic oligonucleotides is ion-pair reversed-phase high-performance liquid chromatography–electrospray ionization mass spectrometry (IP-RP-HPLC-MS), which is utilized to overcome these limitations [13,18,19,20]. A mobile phase consists of an aqueous solution of an alkylamine (trimethylamine (TEA), tributylamine (TBA), dibutylamine (DBA), and others) that improves chromatographic resolution, which is a fluorinated alcohol (hexafluoro-2-propanol (HFIP)). The latter promotes oligonucleotide ionization in the electrospray ionization (ESI) source and, as a result, increases the sensitivity of the method [20,21,22,23].

The IP-RP-HPLC-MS method offers good selectivity, enables the identification and quantification of coeluting impurities, and detects contaminants that are not detectable by more conventional methods. Thus, the IP-RP-HPLC-MS method can provide accurate and reproducible results suitable for the routine quality control of synthetic oligonucleotide therapeutic agents.

## 2. Results and Discussion

Oligonucleotides are complex analytical entities. For example, the complexity of nusinersen is attributable to the replacement of all of its phosphodiester bonds with phosphorothioate bonds, which makes it a heterogeneous mixture comprising 2^17^ variants of diastereoisomers. Each phosphorothioate linkage introduces a chiral phosphorus center, and in long-chain oligonucleotides, results in highly complex diastereomeric mixtures that cannot usually be completely resolved and quantitatively characterized at the level of individual diastereomers with IP-RP-HPLC-MS [24]. Nevertheless, IP-RP-HPLC coupled with high-resolution or ultra-high-resolution mass spectrometry remains an effective and frequently employed approach for distinguishing the main product from closely related impurities, and for monitoring the overall chromatographic profile of the sample [6,7,8]. In addition, IP-RP-HPLC-MS may provide indirect information on the stereochemical heterogeneity of the sample, although its ability to reliably characterize individual diastereomers is limited.

### 2.1. Preliminary Selection of HPLC Conditions

At the first stage of the method development, the results of the chromatographic development were visually assessed for adherence to the following criteria:

- Achieving a smooth baseline with no loss of selectivity.
- Absence of artifact peaks in the baseline for the blank solution.
- Consistency in the chromatographic profile within a single test series and across multiple runs.

In the final stages of development, these criteria were expanded to include the nusinersen peak asymmetry, signal-to-noise (S/N) ratio, and chromatographic resolution between XIC (extracted ion chromatogram) peaks of nusinersen and its *n* − 2 impurities.

### 2.2. Selecting a Chromatography Column

Originally, the pair of HPLC columns from Waters were evaluated: ACQUITY UPLC C18 and ACQUITY Premier BEH Oligonucleotide. The columns were compared in terms of signal stability, retention time stability, chromatographic resolution, and operating pressure. It was observed that oligonucleotides may undergo strong adsorption on certain chromatographic columns, such as ACQUITY UPLC C18, which may result in increased peak area variability between injections and a higher relative standard deviation. Additionally, increasing the column temperature to 85 °C could reduce this adsorption. It was found that the use of an ACQUITY Premier Oligonucleotide BEH C18 column may reduce oligonucleotide adsorption at elevated temperatures.

**Figure 1:**
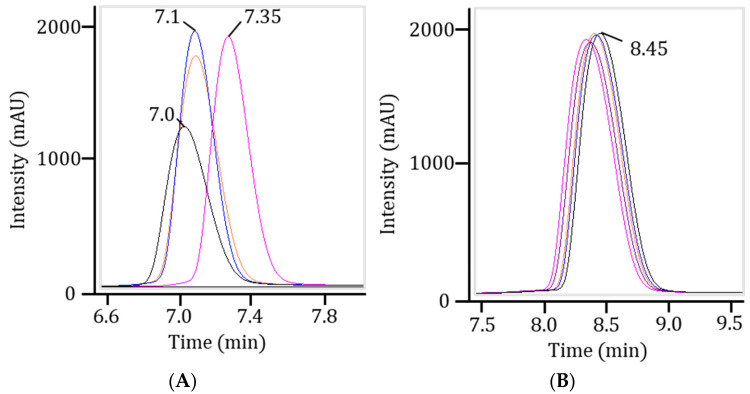
The UV chromatograms of nusinersen samples obtained from the ACQUITY UPLC C18 (**A**) and ACQUITY Premier BEH C18 Oligonucleotide (**B**) columns during the reproducibility test. Note: Wavelength of 260 nm.

It was determined that the originally selected ACQUITY UPLC C18 column showed less reproducibility across runs of the same sample compared to the ACQUITY Premier BEH Oligonucleotide column (Figure 1), likely due to excess analyte adsorption. The latter also showed better separation efficiency and chromatographic resolution between sample components (Table 1). A more concentrated mobile phase was used to further reduce oligonucleotide adsorption (the 8.6 mM TEA, 100 mM HFIP phase was substituted with the 15 mM TEA, 400 mM HFIP phase). Additionally, this mobile phase reduced the average operational pressure in the system from over 500 to 300 bar while maintaining the method’s sensitivity.

In addition, the ACQUITY Premier BEH C18 column can be operated at higher temperatures, which are frequently recommended for oligonucleotide analysis [25,26]. Based on these results, the ACQUITY Premier BEH C18 Oligonucleotide column has been selected for further use.

### 2.3. MS Conditions Optimization

Mass spectrometry of macromolecules, such as proteins and synthetic oligonucleotides, is characterized by relative instability even with mild ionization, a tendency to form adducts with mobile phase components, and the complexity of the resulting spectra. Therefore, the development of such an analysis requires careful optimization of ionization conditions, ion transmission, and proper detection.

**Figure 2:**
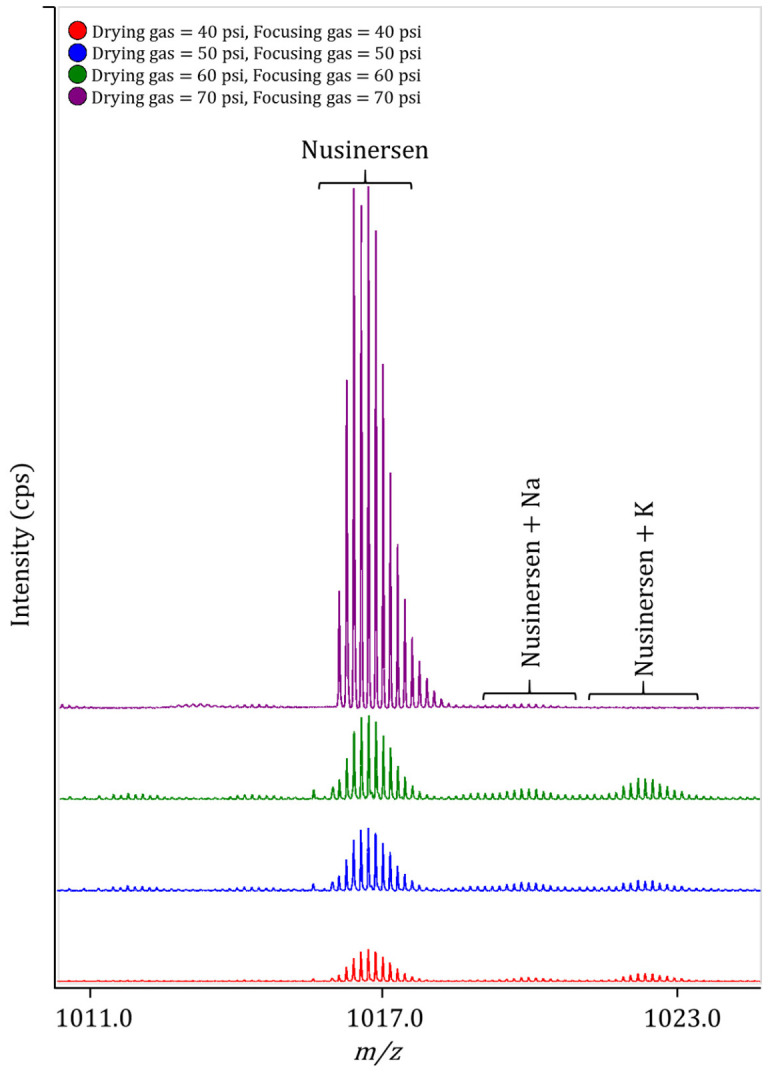
The effect of drying gas and focusing gas on the intensity of nusinersen and its Na- and K-adducts ions (M-7H)^7−^.

First, during the optimization of ionization, we varied the ESI needle voltage to maximize the signal intensity. Finally, this parameter was set to 4500 V. The pressure of the drying and focusing gas varied from 10 to 70 pounds per square inch (psi). At pressures below 40 psi, the required evaporation efficiency was not achieved. At 70 psi of drying and focusing gas, an optimal intensity was obtained with a minimum intensity of adduct ions (Figure 2).

**Figure 3:**
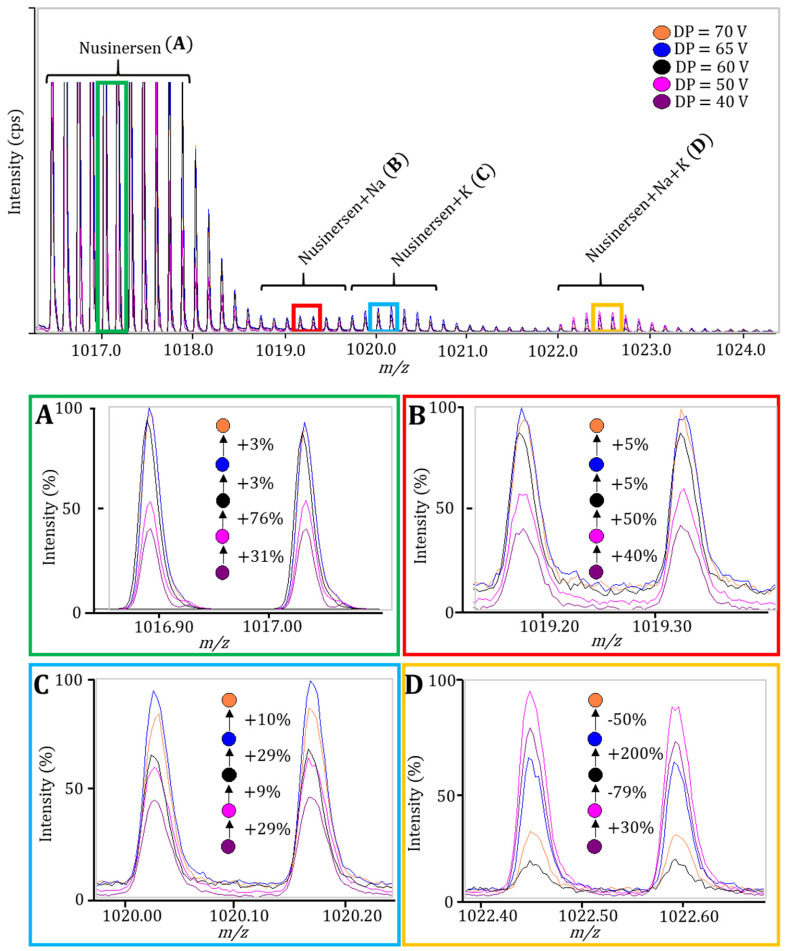
Mass spectra of the nusinersen ion (M-7H)^7−^ and its corresponding adducts show the increase in signal intensity (normalized) with the changes in DP from 40 to 70 V. Note: Influence of declustering potential (DP) on normalized signal intensities for: nusinersen ion (M-7H)^7−^ (**A**), sodium adduct (**B**), potassium adduct (**C**), and combined adduct signal (**D**). Data represent the percentage change in intensity relative to the preceding DP value.

Other combinations of gas pressures were also tested but did not show significant advantages compared to the options described above. Then, the declusterization potential was tested; it is used to further desolvate and purify ions from adducts. This potential was varied from 40 to 70 V, and the selection was guided by maximizing the intensity of desired peaks and minimizing the intensity of unwanted signals from adducts. An optimal value of 60 V was determined (Figure 3). This level demonstrated the maximum difference in increasing the signal of the nusinersen from one side and the signal from its adducts from another.

**Figure 4:**
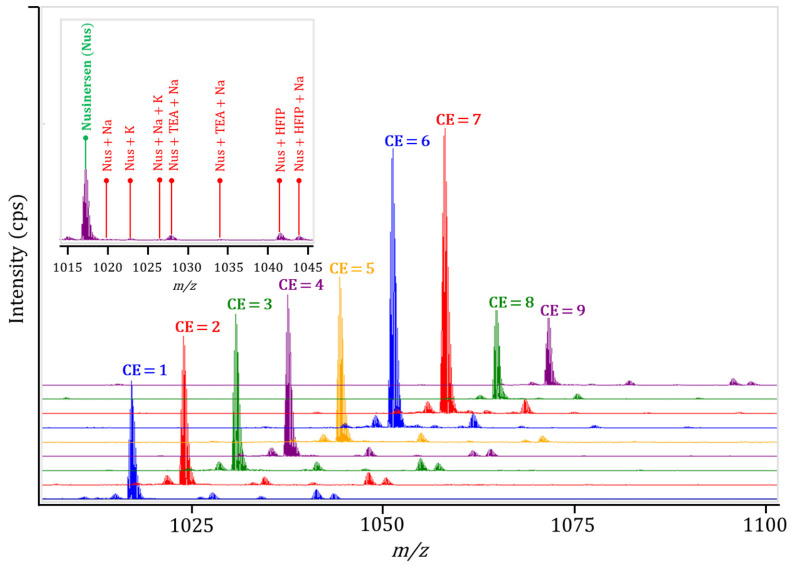
Mass spectra of the nusinersen ion (M-7H)^7−^ and its corresponding adducts with CE varying from 1 to 9 V. Although CE = 6 and CE = 7 provided very similar responses, CE = 7 was selected because it gave a slightly higher nusinersen signal; however, both conditions can be considered practically equivalent. Note: The peak intensity of the nusinersen ion (M-7H)^7−^ for CE = 6 is 26,300 cps, and for CE = 7 the intensity value is 26,700 cps.

At the next stage, collision energy (CE) was varied from 1 to 9 V, aiming for a balance between maximizing signal intensity from nusinersen and minimizing the signal from adducts in the absence of detectable fragmentation. The optimal CE was determined to be 7 V (Figure 4).

### 2.4. Data Collection and Processing

Mass spectra deconvolution and compound identification were performed with Byos software v.5.2.31 (Protein Metrics Inc., Cupertino, CA, USA). TIC and XIC chromatograms, the amount of product- and process-related impurities, and the purity of nusinersen were processed using the Analytics module of Sciex OS software v.3.0.0 and 3.1.6 (Sciex, Framingham, MA, USA).

**Figure 5:**
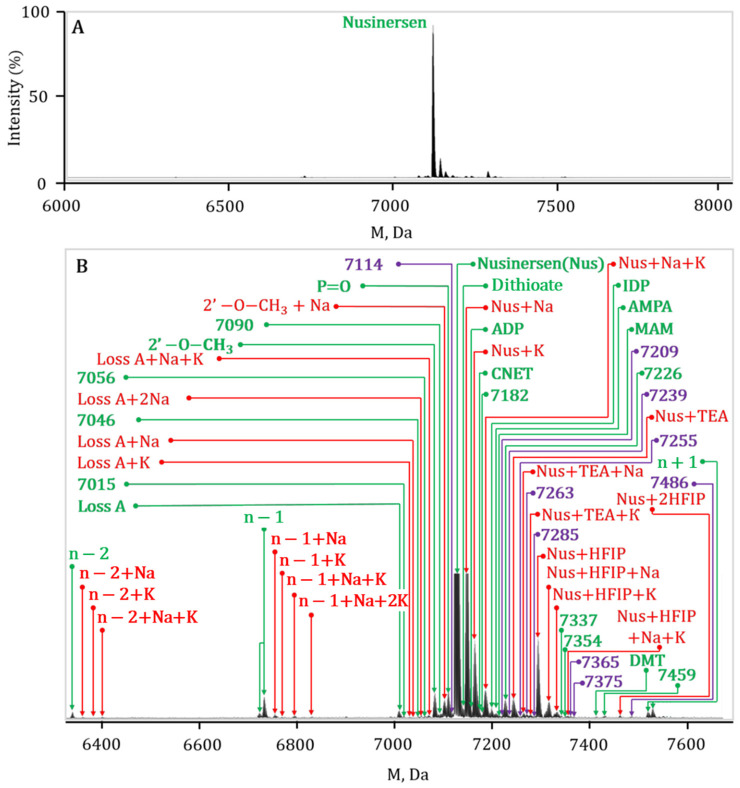
Deconvoluted mass spectrum of nusinersen and its main impurities; (**A**) general view; (**B**) annotated detailed view. The main identified and unidentified impurities are highlighted in green, the adducts are highlighted in red, the unidentified artifacts are highlighted in violet.

Gross formulae and molecular masses of known impurities were calculated using ACD Labs software Version 2025. In addition to easily identifiable nusinersen impurities, which differ from it in retention time, and obvious adducts formed by components of mobile phases (sodium, potassium, TEA, HFIP, and their combinations), a number of ion signals from unidentified components that coelute with nusinersen are always present in the samples. Therefore, their assessment requires special attention (Figure 5).

**Figure 6:**
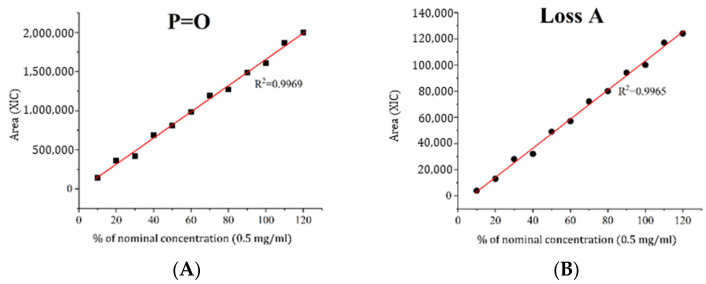

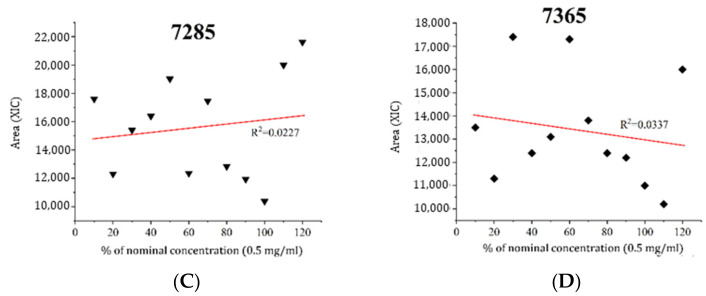
The dependence between the percentage of nominal concentration and the impurities peak area (XIC). (**A**,**B**) The identified impurities; (**C**,**D**) the unidentified artifacts.

First, the nature of these candidates was assessed by examining the linearity of their XIC signals. No linear correlation between signal intensity and content was observed for the artifacts. Additionally, in some instances, a significant shift in the slope of the linearity graphs was noted for the artifacts when compared with the identified impurities of similar amounts (Figure 6).

**Figure 7:**
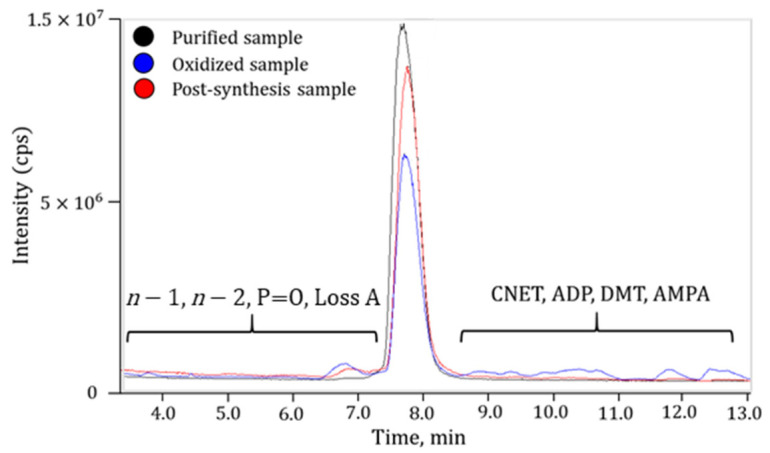
The TIC chromatograms of the purified sample, the post-synthesis sample, and the oxidized sample.

Secondly, to further discriminate other artifacts from impurities, the content of impurities in nusinersen, in a force-oxidized nusinersen (0.05% hydrogen peroxide for 3 h at 30 °C), and in nusinersen without any purification after synthesis were compared (Figure 7). It was hypothesized that neither oxidation nor purification would influence the content of the artifact impurities (Table 2).

During the purification process, the relative concentration of most impurities was reduced, while the artifact impurities remained unchanged. During oxidation, an increase in certain impurities associated with the oxidation of nusinersen was observed, such as P=O and 7090 (double oxidation). In addition, during oxidation of nusinersen with hydrogen peroxide, an increase in *n* − 1 and *n* − 2 was detected, which can be attributed to partial degradation of the nusinersen molecule. This is also supported by a decrease in the relative concentration of *n* + 1. The concentration of artifact impurities remained stable or varied within a certain range. Based on this, a reliable list of impurities present in nusinersen samples was compiled (Table 3).

According to the EMA [7], non-oligonucleotide process impurities include residual reagents, by-products, and solvents, while product-related impurities originate from starting materials, synthesis, or degradation. Reactive starting-material impurities can be incorporated into the oligonucleotide as single-sugar modifications or positional isomers not removed during purification. From the other side, according to MOE amidite discussions, 2′-O-alkyl side-chain introduction generates 2′-O impurities that are critical starting-material impurities [27]. Summing the above, 2′-O-Me and 2′-O-(2-ethoxyethyl) may be considered as both reagent- and process-/synthesis-related impurities. Considering the dithioate/thioate, EMA recognizes them as an impurity class for phosphorothioate oligonucleotides, with process impurities arising from incomplete reactions; thus, dithioate impurities likely result from side reactions in sulfurization chemistry [7].

### 2.5. Chromatographic Behavior of the Main Impurities

To confirm the ability of the method in estimating the amount of impurities, the response factors of the major nusinersen impurities and their chromatographic behavior were investigated. The determination of the response factor has allowed us to conclude that the signals from nusinersen and its main impurities undergo equivalent changes (Table 4).

**Figure 8:**
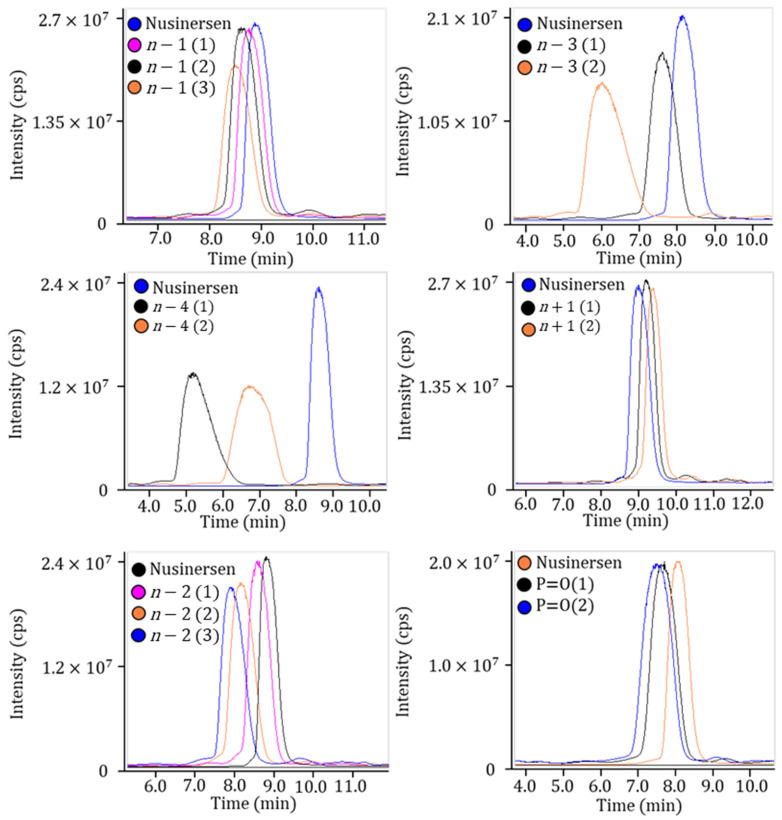
The TIC chromatograms of nusinersen and its main impurities.

Some of the impurities are separated chromatographically from nusinersen, while others coelute with it. It was noted that retention time depends on the position of the modified group in the nusinersen structure and could vary even within the same group of impurities (Figure 8).

The chromatographic behavior of nusinersen and its impurities under ion-pair reversed-phase conditions is determined not only by nucleotide composition but also by the spatial arrangement and positional context of structural variations within the oligonucleotide chain.

The retention characteristics of impurities differ substantially, reflecting conformational and solvent-accessibility effects. The resolution of distinct P=O positional isomers, despite identical molecular mass and overall sequence, illustrates the importance of structural localization. A P=S to P=O substitution at a terminal position is fully exposed to the mobile phase and perturbs the hydrophobic ion-pairing layer formed by TEA and HFIP. This disruption reduces the effective hydrophobic surface area and leads to a measurable decrease in retention time. In contrast, an internal P=O modification may be partially shielded by adjacent 2′-O-methoxyethyl groups, which form a relatively hydrophobic microenvironment. As a result, the impact of such an internal defect on global hydrophobicity and retention is less pronounced.

A similar positional dependence was observed for nucleotide deletion impurities (*n* − 1). Terminal truncations predictably decrease retention in an approximately additive manner due to the reduction in the total hydrophobic surface area. However, internal deletions exert a more complex effect. Removal of a nucleotide within the sequence disrupts π–π stacking interactions between adjacent bases, which contribute to the stabilization of a compact hydrophobic core. This perturbation increases local conformational flexibility and enhances solvent exposure of the negatively charged phosphorothioate backbone. Consequently, internal deletions may produce a disproportionately large reduction in retention compared with terminal losses of equivalent mass.

For *n* − 2, *n* − 3, and *n* − 4 impurities, the chromatographic behavior demonstrates pronounced sequence-context dependence. Deletion of hydrophobic segments, such as the 3′-terminal guanine stretch (3′-GG), markedly decreases affinity for the stationary phase and shifts the hydrophobic–hydrophilic balance. Overall, these observations confirm that chromatographic separation of nusinersen-related impurities reflects structural and conformational variations, underscoring the necessity of high-resolution LC–MS for accurate impurity profiling.

**Figure 9:**
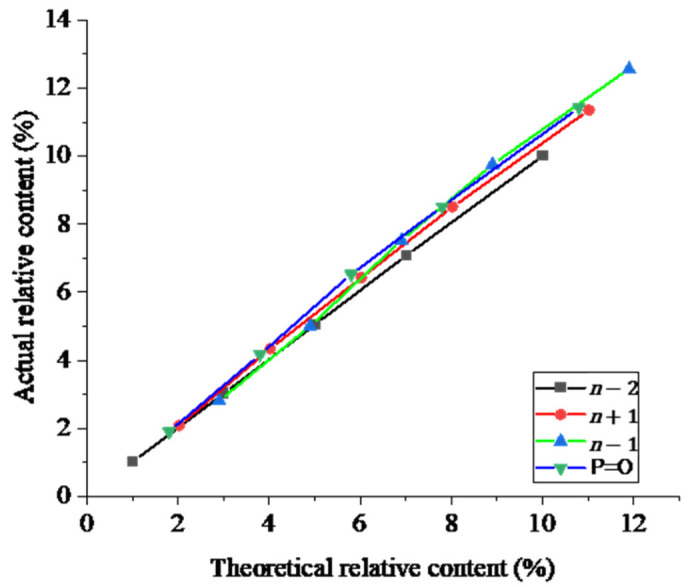
The correlation between the actual content of an impurity and its expected value.

To assess the accuracy of the method, nusinersen spiked with equimolar mixes of various impurities at concentrations of 1, 3, 5, 7, and 10% (P=O, *n* − 2, *n* − 1, and *n* + 1) was analyzed. As the concentration of impurities increased, the area of their peaks demonstrated a proportional increase (Figure 9 and Table 5), thereby confirming the linearity and accuracy of the method.

### 2.6. The Impact of Sample Preparation

**Figure 10:**
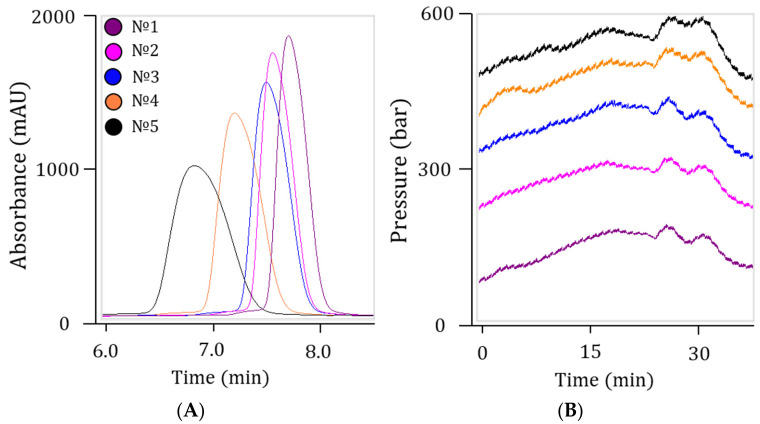
The effect of consecutive analysis of the nusinersen samples without sample preparation on chromatographic separation (**A**) and system pressure (**B**). The numbers indicate the injection replicates.

The necessity of sample preparation was due to the increase in system pressure and the decrease in chromatographic resolution during multiple injections (Figure 10). Additionally, when analyzing nusinersen synthesis and purification semi-products without prior sample preparation, a complete loss of chromatographic equilibrium was frequently observed, accompanied by unpredictable drift of retention time and a multiple increase in peak width. Even after purification, the nusinersen solution may contain residual buffer solutions, reagent byproducts, mechanical particles, and other substances that could negatively affect the LC-MS system and distort analysis results.

During the comparison, it was found that there was no statistically significant difference in the relative amount of impurities between the samples with and without sample preparation (Table 6). The reported results were calculated based on three independent sample preparations carried out on separate days. Each preparation was analyzed in triplicate injections, resulting in a total of nine measurements per evaluated parameter.

### 2.7. Method Validation

The method was validated in terms of specificity, linearity, precision, accuracy and robustness.

#### 2.7.1. Specificity

The specificity of the method was verified using both negative and positive control samples (Figure S1). A placebo sample was used as a negative control, while samples of the most prominent oligonucleotide impurities, including P=O, *n* − 1, *n* − 2, and *n* + 1 impurities with different modifications of the bond and deleted site location, were used as positive controls. To confirm the equivalence between changes in the signal response of oligonucleotides and their major impurities, and changes in their proportions in samples, a response factor was determined (Table 6), demonstrating proportional relationships between increases in peak areas for the studied impurities and increases in their concentrations.

#### 2.7.2. The Limit of Detection (LoD) and the Limit of Quantitation (LoQ), and Linearity

Model solutions of therapeutic oligonucleotide were prepared in the concentration range of 9.6–1.0 × 10^−5^ mg/mL. The coefficients of determination (R^2^) for the peak areas of TIC (total ion current) and XIC (extracted ion current) were calculated, with both values exceeding 0.99. For the nusinersen XIC, the limits of detection (LoD) were set at 2.5 × 10^−5^ mg/mL, and the limits of quantitation (LoQ) at 4.9 × 10^−5^ mg/mL (Table S1 and Figures S2 and S3).

#### 2.7.3. The Linearity of the MS Signal from Impurities

The linearity of the analytical method was also assessed using XIC chromatograms for each impurity (Table 7). It was found that, at low test sample concentrations (up to 0.2 mg/mL), the method exhibited decreased sensitivity to some impurities. Therefore, the analytical range of the method was adjusted to 0.2–1.0 mg/mL to minimize the risk of false-negative results for minor impurities (Table S3).

#### 2.7.4. Repeatability

The repeatability was assessed in a single analytical run on six independent samples prepared by one analyst. The relative standard deviation of the area of oligonucleotides and impurities was determined using XIC chromatograms ${RSD}_{XIC}^{Area}$. All impurities with a relative content of more than 1.0% had ${RSD}_{XIC}^{Area}$ values that do not exceed 2.7%. The ${RSD}_{XIC}^{Area}$ values for peaks with a relative content of less than 1.0% vary from 2.5% to 7.3%, which do not exceed the maximum allowable value of 15.0% [28]. Repeatability was positively assessed with regard to relative areas (Table S4).

#### 2.7.5. Reproducibility

The reproducibility was estimated using the Agilent 1260 Infinity II Bio with the Sciex X500B system, with sample preparation carried out by two analysts on different days (Table S5). The ${RSD}_{XIC}^{Area}$ values for peaks with a relative content greater than 1.0% are less than 4.0%, which is the maximum allowable value. Impurities with a relative content between 0.2% and 1.0% have the ${RSD}_{XIC}^{Area}$ ranging from 1.9% to 7.9%, which is also less than the maximum allowable limit of 8.0% [28].

#### 2.7.6. Robustness—Using Different HPLC-MS Systems

**Figure 11:**
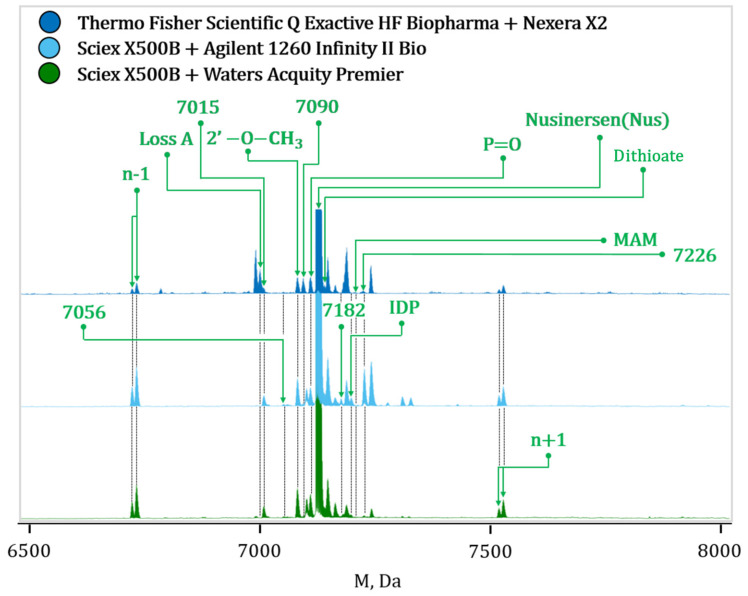
Comparison of the nusinersen mass spectra after deconvolution from different LC-MS instruments. The adducts and artifact signals are not annotated.

The developed method was used to analyze nusinersen samples using the Agilent 1260 Infinity II Bio with Sciex X500B and Sciex OS v.3.0.0 software, Waters Acquity Premier with Sciex X500B and Sciex OS v.3.1.6 software, and Shimadzu Nexera X2 with Thermo Fisher Scientific Q Exactive HF Biopharma with Chromeleon v.7.2.10 software systems. The chromatography and mass spectrometry operating conditions were similar. The ionization parameters of the Q Exactive HF Biopharma mass spectrometer differed slightly from those of the Sciex X500B instrument, but the essential parameters were consistent. The amounts of nusinersen and its impurities were determined using Byos software (Figure 11, Table 8).

The main differences in the collected data can be attributed to the different operational principles of the devices and architecture of the ion sources. The lower relative content of *n* − 1 and *n* − 2 observed with the Shimadzu Nexera X2 + Thermo Fisher Scientific Q Exactive HF Biopharma system may reflect differences in ionization/response characteristics and relative detection of these species. The lack of oxygen in the ion source of the Shimadzu Nexera X2 + Thermo Fisher Scientific Q Exactive HF Biopharma system also reduces the oxidation of oligonucleotides, slightly lowering the level of P=O. Overall, a satisfactory level of consistency was observed in the results obtained using the three different HPLC-MS systems. This suggests the robustness of the method, although there were slight differences in the relative concentrations of impurities.

The method demonstrated adequate robustness with respect to small deliberate variations in critical parameters. Column temperature variations within ±2 °C and ion source temperature variations within ±10 °C did not result in statistically significant changes in impurity profiling. The mobile phase was stable for up to four days when stored at 25 ± 2 °C. Prepared nusinersen samples were stable for at least 24 h at 2–8 °C (Tables S6 and S7).

## 3. Materials and Methods

Method development and optimization were performed using the Agilent 1260 Infinity II Bio (Waldbronn, Germany) coupled to the Sciex X500B system. The validation was performed using a Sciex X500B mass spectrometer equipped with two HPLC systems: the Agilent 1260 II Bio and Waters Premier. Both systems fullfilled the validation criteria, but Waters Premier demonstrated a more consistent results with a relatively low flow rate used in our method (0.2 mL/min). Considering the latter, it was chosen for the subsequent routine quality control of nusinersen. The Shimadzu Nexera X2–Thermo Q Exactive HF Biopharma system (Canby, OR, USA) was used primarily to assess method robustness and inter-platform comparability.

### 3.1. Reagents

LC-MS-grade triethylamine was purchased from Sigma-Aldrich, a division of Merck (Saint Louis, MO, USA) and Honeywell/Fluka (Seelze, Germany). Hexafluoro-2-propanol was purchased from P&M Invest (Moscow, Russia). LC-MS-grade methanol was purchased from Merck (Darmstadt, Germany). The water for the preparation of mobile phases and sample preparation was purified using a Milli-Q IQ 7000 system (Merck, Molsheim, France).

### 3.2. Samples

The antisense oligonucleotide nusinersen contains phosphorothioate bonds instead of phosphate bonds and has 2′-hydroxy groups of ribofuranosyl rings substituted with 2′-O-2-methoxyethyl groups. Nusinersen and its main impurities were manufactured by Generium JSC using solid phase synthesis (Table 9).

### 3.3. Sample Preparation

The nusinersen samples had a concentration of 2.4 mg/mL. Before analysis, the samples were diluted with water to a nominal concentration of 0.5 mg/mL and then filtered using Merck Millipore syringe filters (pore diameter of 0.22 µm). After this, the buffer was changed to water using Amicon Ultra 3K ultrafiltration modules from Merck (Darmstadt, Germany). An amount of 200 μL of the filtrate was transferred to the ultrafiltration cells, followed by the addition of 300 μL of water. The modules were then subjected to centrifugation (10 min; 10,000× *g*). Following centrifugation, approximately 100–150 μL of concentrate remained in the ultrafiltration cell. The filtrate was then drained from the test tube, and water was added to the ultrafiltration module to an approximate volume of 500 μL. This process was repeated in triplicate. After four ultrafiltration cycles, the volume of concentrates was increased to an approximate value of 200 μL with water.

### 3.4. Instruments

The Agilent 1260 Infinity II Bio-inert (Waldbronn, Germany), Waters Acquity Premier (Singapore), and Shimadzu Nexera X2 (Canby, OR, USA) high-performance liquid chromatography (HPLC) systems were used in the study. The liquid chromatography systems were equipped with quaternary (Agilent) or binary (Waters and Shimadzu) pumps, a degasser, a thermostat, an autosampler, and a UV detector. The Agilent 1260 Infinity II Bio-inert and the Waters Acquity Premier were coupled to a Sciex X500B QTOF mass spectrometer (Singapore), and the Shimadzu Nexera X2 was coupled to a Thermo Fisher Scientific Q Exactive HF (Bremen, Germany). Both mass spectrometers were equipped with heated electrospray ionization sources.

### 3.5. Chromatographic Column and Mobile Phases

The ACQUITY Premier Oligonucleotide BEH C18, 130 Å, 1.7 µm, 2.1 × 150 mm (Waters, Milford, MA, USA, cat. no. 186009486), and the ACQUITY UPLC BEH C18, 1.7 µm, 2.1 × 100 mm (Waters, Milford, MA, USA, cat. no. 186002352), were used in this study. The thermostat temperature was maintained at 85 °C. The mobile phase A was prepared in two variations: 8.6 mM TEA and 100 mM HFIP, and 15 mM TEA and 400 mM HFIP. Mobile phase B consisted of methanol.

### 3.6. LC-MS Conditions

The mobile phase flow rate was 0.2 mL/min. Sample injection volume was 5 µL (2.5 µg), and the column thermostat was set to 85 °C. The elution was carried out in gradient mode according to the program presented in Table 10.

Flow was diverted to waste during the first two minutes of the LC-MS method to prevent contamination of the ion source with trace amounts of salts from the samples. Both mass spectrometers used electrospray ionization at atmospheric pressure in negative ionization mode.

#### 3.6.1. MS Conditions for Sciex X500B

The needle voltage was 4500 V, drying gas flow and focusing gas flow were 70 psi, source temperature was maintained at 350 °C, collision cell gas pressure (CAD) was 7 psi, declustering potential (DP) was 60 V, collision energy (CE) was 7 V, and scan range was 600–4000 *m*/*z*. The mass spectrometer was operated in Intact Protein mode.

#### 3.6.2. MS Conditions for Q Exactive HF

Because the Sciex X500B QTOF and Thermo Fisher Scientific Q Exactive HF Orbitrap platforms differ in source architecture, ion-transfer settings, and acquisition principles, the MS parameters were not transferred directly between instruments but only aligned at a functional level.

The spray voltage was 4300 V, capillary temperature was 400 °C, sheath gas was 40, aux gas was 10, probe heater temperature was 325 °C, S-Lens RF-level was 80, in-source CID was 0 eV, resolution was 120,000, maximum IT was 200 ms, and scan range was 800–4000 *m*/*z*. The mass spectrometer was operated in Intact Protein mode.

## 4. Conclusions

The developed method for the determination of the purity of nusinersen using high-performance liquid chromatography combined with high-resolution mass spectrometry has been an important tool for analyzing the purity of both the oligonucleotide drug substance and drug product. This method has allowed for routine purity monitoring, which has provided us with important information about stability, stress degradation, and the effects of purification in the development of manufacturing processes. The method has enabled us to collect data that have had a significant impact on the techniques developed for the synthesis, purification, and selection of storage conditions. These findings have led to the optimization of each stage of production and to an understanding of degradation pathways, reducing the risks associated with the storage of the final product. Ultimately, the development of this technique has contributed to the production of the final drug with a high level of purity.

Although the developed method is specific to nusinersen and cannot be directly transferred to other oligonucleotide drugs, the general LC-MS workflow and its nuances applied here may serve as a useful basis for quality control method development for other synthetic oligonucleotides in pharmaceutical and other QC laboratories.

## Figures and Tables

**Table 1:** The number of theoretical plates (*N*) and resolution between the main peak of nusinersen and the *n* − 2 impurity (R_Nus vs n-2_) for ACQUITY UPLC C18 and ACQUITY Premier BEH C18 Oligonucleotide columns.

Chromatographic Column	*N*	R_Nus vs n-2_
ACQUITY UPLC C18	1500–1900	0.8–0.9
ACQUITY Premier BEH C18 Oligonucleotide	4900–5200	2.9–3.3

**Table 2:** The relative impurity content of the post-synthesis sample, the purified nusinersen, and the force-oxidized sample.

Substance/Impurity	The Relative Impurity Content (%)		
	Post-Synthesis Sample	Purified Sample	Oxidized Sample
*n* − 2	0.288	0.014	0.316
*n* − 1	1.853	0.897	1.900
Loss A	0.605	0.456	0.551
7015	0.195	0.123	0.204
7046	0.221	0.174	0.279
7056	0.176	0.122	0.213
2′-O-Me	1.414	1.112	1.174
7090	0.199	0.179	3.105
P=O	1.518	0.755	21.423
7114 *	0.383	0.390	0.389
Nusinersen	82.764	90.444	59.270
Ditioate	0.611	0.471	0.834
ADP	0.284	0.011	0.295
CNET	0.619	0.003	0.715
7182	0.273	0.001	0.331
IDP	0.521	0.199	0.667
AMPA	0.217	0.020	0.209
MAM	0.252	0.190	0.353
7209 *	0.300	0.300	0.295
7226	1.409	0.196	1.512
7239 *	0.233	0.241	0.234
7255 *	0.252	0.245	0.250
7267 *	0.383	0.389	0.388
7285 *	0.280	0.290	0.278
7337	0.374	0.258	0.375
7354	0.512	0.015	0.512
7365 *	0.238	0.243	0.242
7375 *	0.296	0.212	0.312
DMT	0.267	0.011	0.229
7459	0.339	0.295	0.339
7547	0.214	0.009	0.214
7486 *	0.114	0.122	0.110
*n* + 1	1.898	1.039	1.708
8275	0.138	0.124	0.534
dimer	0.360	0.450	0.240

* The artifact impurities.

**Table 3:** The identified oligonucleotide compounds after filtering out artifacts and adducts.

Name	Monoisotopic Mass (Da)	Description	Origin
Nusinersen	7122.24	Full-length oligonucleotide (*n*)	Main product
*n* − 1	6703.21 6719.20 6729.20 6728.22	The total content of impurities with loss of one nucleotide (the underlined masses were experimentally observed above or near the LOQ; all listed masses are monitored in quality control).	Process-/synthesis-related impurities
*n* − 2	6284.15 6300.14 6310.14 6309.15 6316.14 6326.13 6325.15 6336.13 6335.14 6334.16	The total content of impurities with the loss of two nucleotides (the underlined mass was experimentally observed above the LOQ; all listed masses are monitored in quality control)	Process-/synthesis-related impurities
P=O	7106.28	One thiophosphate bond is oxidized to a phosphate ester	Process-/synthesis-related impurities
Loss A	6988.22	An oligonucleotide with a cleavage of one adenine	Presumably analysis-related signal
2′-O-Me	7078.25	Modification of the 2′-carbohydrate group	Process-/synthesis-related impurities
*n* + 1	7515.35 7516.34 7525.35 7541.34	An oligonucleotide with one additional nucleotide (the underlined masses were experimentally observed above or near the LOQ; all listed masses are monitored in quality control)	Process-/synthesis-related impurities
N^2^-isobutyryl-2,6-diaminopurine (IDP)	7195.33	Modification of the purine base	Process-/synthesis-related impurity
N-methylacetamidomethyl (MAM)	7204.29	Reagent-related impurity	Reagent-related impurity
N^3^-(2-cyanoethyl)thymine (CNET)	7171.25	Modification of thymine	Process-/synthesis-related impurity
N^2^-acetyl-2,6-diaminopurine (ADP)	7157.20	Modification of the purine base	Process-/synthesis-related impurity
2′-O-(2-ethoxyethyl)/Dithioate	7139.15	Modification of the 2′-carbohydrate group; under the selected LC-MS conditions, the XIC signal in this region was interpreted jointly because it may include contributions from both closely related species, whose similar masses and chromatographic behavior limited reliable differentiation	Process-/synthesis-related impurity (potentially originating from a critical impurity in the starting material)
DMT	7426.37	Full-length oligonucleotide (*n*) with dimethoxytrityl group	Process-/synthesis-related impurity
3-(3-acetyl-4-methylpyrimidine-2-oh-6-yl)-2-aminoimidazole (AMPA)	7205.45	Modification of the pyrimidine base	Process-/synthesis-related impurity
Dimer	14,252.10	The product of decomposition may form in small amounts under the influence of light	Presumably analysis-/handling-related signal
7226, 7182, 7459, 7354, 7015, 7337, 7090, 8275, 7547, 7046, 7056	Unidentified impurities with amounts above the limit of quantitation. They have been marked based on their monoisotopic mass rounded to the nearest integer		Specified unidentified impurities

**Table 4:** The relative content of major impurities/nusinersen and its response factors.

Name	Relative Content (%)	Absolute TIC Peak Area (cps)	Response Factor
*n* − 1(1)	89.99	7.10 × 10^8^	0.90
*n* − 1(2)	91.78	7.49 × 10^8^	0.97
*n* − 1(3)	90.72	7.48 × 10^8^	0.96
Nusinersen	95.44	7.41 × 10^8^	1.00
*n* − 2(1)	91.03	7.50 × 10^8^	0.96
*n* − 2(2)	91.46	8.23 × 10^8^	1.06
*n* − 2(3)	89.31	7.11 × 10^8^	0.89
Nusinersen	95.62	7.45 × 10^8^	1.00
*n* − 3(1)	92.05	7.39 × 10^8^	0.86
*n* − 3(2)	96.45	8.60 × 10^8^	1.05
Nusinersen	95.57	8.24 × 10^8^	1.00
*n* − 4(1)	95.64	6.79 × 10^8^	1.07
*n* − 4(2)	92.77	6.43 × 10^8^	0.99
Nusinersen	96.21	6.28 × 10^8^	1.00
*n* + 1(1)	83.22	6.70 × 10^8^	0.88
*n* + 1(2)	83.00	6.15 × 10^8^	0.81
Nusinersen	83.22	6.61 × 10^8^	1.00
P=O(1)	85.12	9.80 × 10^8^	1.04
P=O(2)	82.93	8.85 × 10^8^	0.92
Nusinersen	95.89	8.34 × 10^8^	1.00

**Table 5:** Recovery factor for the main impurities of nusinersen.

Impurity	Actual Content of an Impurity, C_fact_ (%)	Expected Content of an Impurity, C_theor_ (%)	C_fact_ − C_theor_ (%)	Recovery Factor (%)
*n* − 2	1.02	1.01	0.01	101
	3.02	3.01	0.01	100
	5.05	5.01	0.04	101
	7.08	7.01	0.07	101
	10.01	10.01	0.00	100
*n* + 1	2.08	2.02	0.06	103
	4.34	4.02	0.32	108
	6.43	6.02	0.41	107
	8.51	8.02	0.48	106
	11.36	11.02	0.33	103
*n* − 1	2.80	2.90	0.10	97
	5.01	4.90	0.11	102
	7.53	6.90	0.63	109
	9.75	8.90	0.85	110
	12.57	11.90	0.67	106
P=O	1.89	1.80	0.09	105
	4.17	3.80	0.37	110
	6.53	5.80	0.73	113
	8.52	7.80	0.72	109
	11.42	10.80	0.62	106

**Table 6:** The impact of sample preparation on the relative content of nusinersen and its impurities.

Substance/ Impurity	Relative Content (%)	
	Without Sample Preparation	With Sample Preparation
Nusinersen	92.700 ± 0.104	92.653 ± 0.118
*n* − 2	0.014 ± 0.001	0.014 ± 0.007
*n* − 1	0.919 ± 0.070	0.896 ± 0.034
*n* + 1	1.065 ± 0.037	1.106 ± 0.040
P=O	0.774 ± 0.091	0.697 ± 0.120
Dithioate	0.483 ± 0.013	0.479 ± 0.024
2′-O-Me	1.140 ± 0.033	1.158 ± 0.037
Loss A	0.467 ± 0.018	0.452 ± 0.023
CNET	0.003 ± 0.002	0.005 ± 0.004
ADP	0.011 ± 0.001	0.012 ± 0.001
IDP	0.204 ± 0.036	0.236 ± 0.054
AMPA	0.020 ± 0.003	0.021 ± 0.006
MAM	0.195 ± 0.001	0.196 ± 0.007
DMT	0.011 ± 0.002	0.015 ± 0.001
Dimer	0.461 ± 0.055	0.484 ± 0.052
7015	0.126 ± 0.003	0.127 ± 0.004
7046	0.178 ± 0.003	0.180 ± 0.007
7056	0.125 ± 0.004	0.112 ± 0.008
7090	0.183 ± 0.011	0.188 ± 0.014
7182	0.001 ± 0.004	0.002 ± 0.006
7226	0.201 ± 0.001	0.212 ± 0.002
7337	0.264 ± 0.004	0.261 ± 0.005
7354	0.015 ± 0.002	0.020 ± 0.001
7459	0.302 ± 0.005	0.333 ± 0.008
7547	0.009 ± 0.009	0.019 ± 0.010
8275	0.127 ± 0.011	0.122 ± 0.012

**Table 7:** The coefficient of determination for some impurities.

Substance/Impurity	R^2^
Nusinersen	0.9948
*n* − 2	0.9974
*n* − 1	0.9955
*n* + 1	0.9868
P=O	0.9969
Dithioate	0.9669
2′-O-Me	0.9906
Loss A	0.9965
CNET	0.9936
ADP	0.9774
IDP	0.9875
AMPA	0.9934
MAM	0.9881
DMT	0.9865
Dimer	0.9995
7015	0.9953
7046	0.9958
7056	0.9896
7090	0.9905
7182	0.9673
7226	0.9918
7337	0.9786
7354	0.9886
7459	0.9709
7547	0.9847
8275	0.9778

**Table 8:** Comparison of the relative content (%) of nusinersen and its impurities using various LC-MS systems.

Substance/ Impurity	Waters Acquity Premier + Sciex X500B	Agilent 1260 Infinity II Bio + Sciex X500B	Shimadzu Nexera X2 + Thermo Fisher Scientific Q Exactive HF Biopharma
Nusinersen	94.54	95.28	95.88
*n* − 2	<0.02	<0.02	<0.02
*n* − 1	1.11	1.08	0.56
*n* + 1	0.79	0.75	0.42
P=O	0.71	0.66	0.57
Dithioate	0.52	0.37	0.24
2′-O-Me	0.82	0.62	0.55
Loss A	0.39	0.28	0.20
CNET	<0.02	<0.02	<0.02
ADP	0.02	<0.02	<0.02
IDP	0.24	0.26	0.06
AMPA	0.02	<0.02	<0.02
MAM	<0.02	<0.02	<0.02
DMT	<0.02	<0.02	<0.02
Dimer	0.31	0.32	0.59
7015	0.05	0.04	0.04
7046	0.04	0.02	0.05
7056	0.07	0.05	0.04
7090	0.22	0.10	0.42
7182	<0.02	<0.02	<0.02
7226	<0.02	<0.02	<0.02
7337	0.03	0.03	<0.02
7354	<0.02	<0.02	<0.02
7459	0.03	0.04	0.02
7547	0.02	<0.02	<0.02
8275	0.05	0.08	0.33

**Table 9:** A simplified view of the nucleotide sequence of nusinersen and its impurities synthesized for method development.

Name	Sequence
Nusinersen	mU-mC-A-mC-mU-mU-mU-mC-A-mU-A-A-mU-G-mC-mU-G-G
*n* − 1(1)	mU-mC-A-mC-mU-mU-mU-mC-A-mU-A-A-mU-G-mC-mU-G-×
*n* − 1(2)	××-mC-A-mC-mU-mU-mU-mC-A-mU-A-A-mU-G-mC-mU-G-G
*n* − 1(3)	mU-mC-A-mC-mU-mU-mU-mC-×-mU-A-A-mU-G-mC-mU-G-G
*n* − 2(1)	××-mC-A-mC-mU-mU-mU-mC-A-mU-A-A-mU-G-mC-mU-G-×
*n* − 2(2)	××-××-A-mC-mU-mU-mU-mC-A-mU-A-A-mU-G-mC-mU-G-G
*n* − 2(3)	mU-mC-A-mC-mU-mU-mU-mC-A-mU-A-A-mU-G-mC-mU-×-×
*n* − 3(1)	mU-mC-A-mC-mU-mU-mU-mC-A-mU-A-A-mU-G-mC-××-×-×
*n* − 3(2)	××-××-×-mC-mU-mU-mU-mC-A-mU-A-A-mU-G-mC-mU-G-G
*n* − 4(1)	mU-mC-A-mC-mU-mU-mU-mC-A-mU-A-A-mU-G-××-××-×-×
*n* − 4(2)	××-××-×-××-mU-mU-mU-mC-A-mU-A-A-mU-G-mC-mU-G-G
*n* + 1(1)	mU-mC-A-mC-mU-mU-mU-mC-A-mU-A-A-mU-G-mC-mU-G-G-G
*n* + 1(2)	mU-mC-A-mC-mU-mU-mU-mC-A-mU-A-A-mU-G-mC-mU-G-G-mC
P=O(1)	mU_(P=O)_-mC-A-mC-mU-mU-mU-mC-A-mU-A-A-mU-G-mC-mU-G-G
P=O(2)	mU-mC-A-mC-mU-mU-mU-mC-A-mU_(P=O)_-A-A-mU-G-mC-mU-G-G

Note: ‘×’ or ‘××’ represents the deletion of one nucleotide. A, G, mC, and mU represent adenine, guanine, methylcytosine, and methyluracil, respectively. mU_(P=O)_- nucleotide containing one native phosphodiester linkage.

**Table 10:** The gradient program for Agilent 1260 Infinity II Bio-inert and Waters Acquity Premier/Shimadzu Nexera X2 systems.

Time (min)	Phase B for Agilent 1260 Infinity II Bio-Inert (%)	Phase B for Waters Acquity Premier/Shimadzu Nexera X2 (%)
0.00	27	20
12.00	42	33
16.00	90	90
17.00	90	90
17.01	27	20
32.00	27	20

## Data Availability

The data that support the findings of this study are available from the corresponding author upon reasonable request.

## Supplementary Materials

The following supporting information can be downloaded at: https://www.mdpi.com/article/10.3390/ijms27073301/s1.

## Abbreviations

The following abbreviations are used in this manuscript:

CAD	Collision-Activated Dissociation
CE	Collision Energy
DBA	Dibutylamine
DP	Declustering Potential
EMA	European Medicines Agency
ESI	Electrospray Ionization
FDA	Food and Drug Administration
HFIP	1,1,1,3,3,3-Hexafluoro-2-propanol
HPLC	High-Performance Liquid Chromatography
LC-MS	Liquid Chromatography–Mass Spectrometry
LoD	Limit of Detection
LoQ	Limit of Quantification (or Limit of Quantitation)
QTOF	Quadrupole Time-of-Flight (Mass Spectrometry)
RSD	Relative Standard Deviation
S/N	Signal-to-Noise Ratio
*SMN1*	Survival Motor Neuron 1 (gene)
TBA	Tributylamine
TEA	Triethylamine
TIC	Total Ion Chromatogram
XIC	Extracted Ion Chromatogram
