# Supplementary material for: Development and Validation of an Ion-Pair Reverse-Phase High-Performance Liquid Chromatography–Electrospray Ionization Mass Spectrometry Method for Determination of Purity of Nusinersen for Quality Control of Drug Substance or Drug Product
Source: Int J Mol Sci. 2026 Apr 5;27(7):3301. doi: 10.3390/ijms27073301 (PMC13072881; doi:10.3390/ijms27073301)
Supplement: Supplementary file 1 [file ijms-27-03301-s001.zip › ijms-4215511-supplementary.pdf]

Supplementary Materials

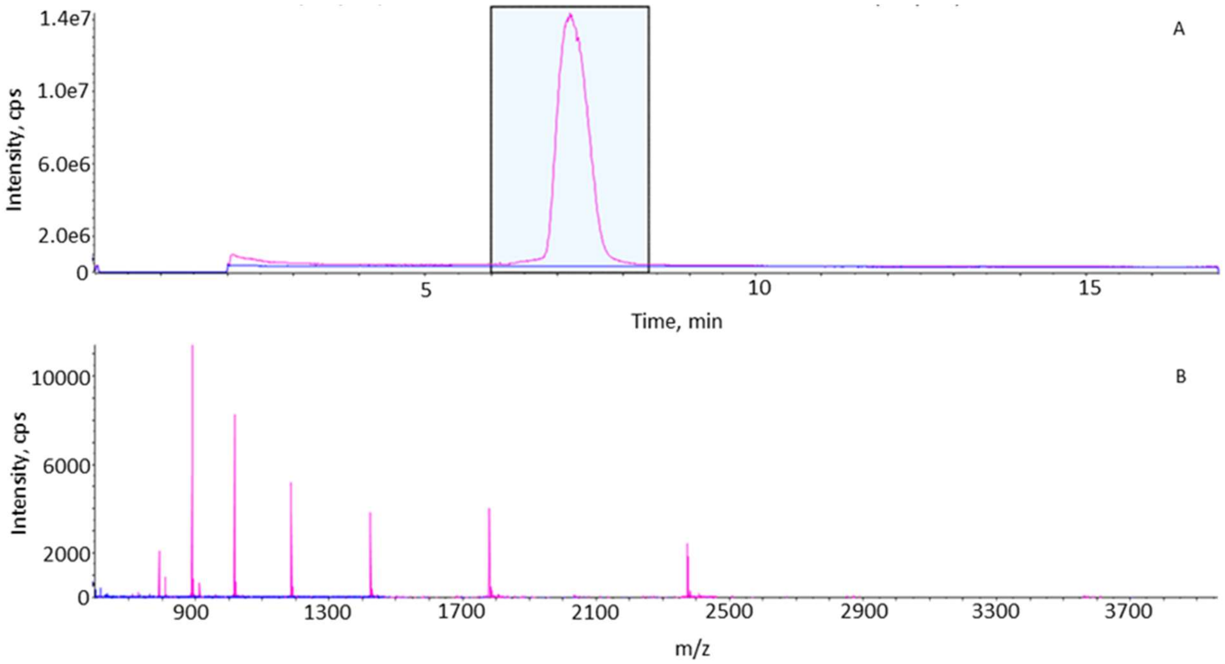

Figure S1. Total ion chromatograms (TICs) (**A**) and mass spectra extracted from the selected region (**B**) for the nusinersen sample (pink) and the blank solution (blue).

Table S1. Dependence of peak area on nusinersen concentration based on XIC chromatograms and corresponding S/N values.

Nusinersen concentration, mg/mL	Peak area, a.u.	Signal-to-noise ratio
0.00000078	5.4	1.0
0.0000016	7.8	0.9
0.0000031	13.2	1.7
0.0000063	28.5	0.9
0.000013	7.2	2.5
0.000025	39.3	3.5
0.00005	119.6	32.5
0.0001	1594.0	151.9
0.0002	6993.0	350.9
0.00039	23083.3	649.8
0.00078	63286.7	1093.1
0.0016	158400.0	1380.9
0.0031	431466.7	2357.5
0.0062	964166.7	3477.3
0.013	1995666.7	4938.6
0.025	4044666.7	7565.3
0.05	6913000.0	9389.5
0.1	12373333.3	11560.0
0.2	21896666.7	22119.9
0.4	39203333.3	27318.3
0.64	69843333.3	32380.4
0.8	98660000.0	35009.1
1.0	112733333.3	35221.6

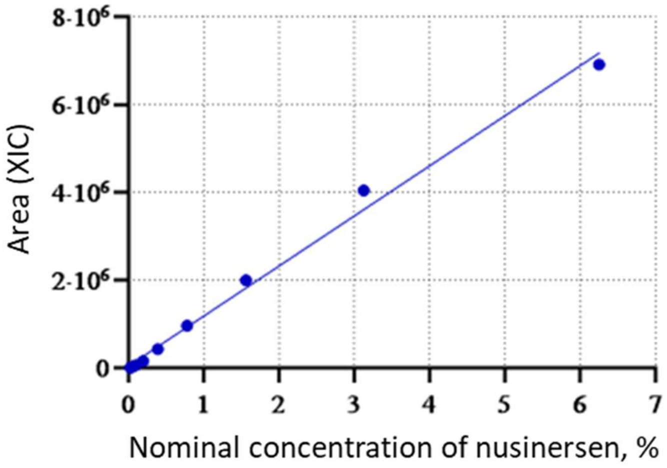

Figure S2. Linear regression plot in the range 0.0002–0.05 mg/mL (corresponding to the expected impurity content in the samples).

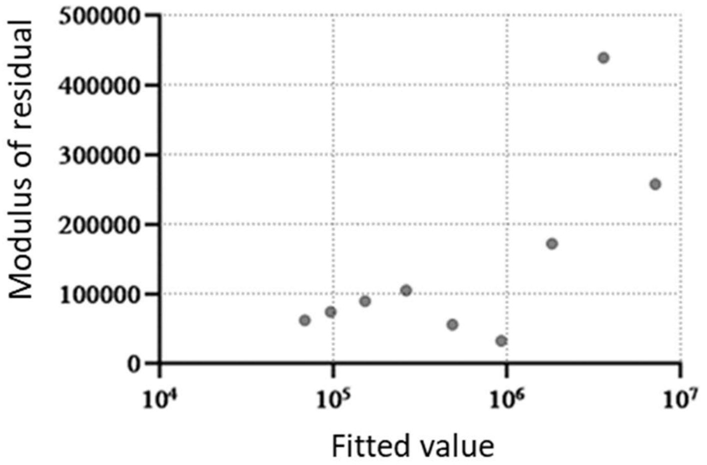

Figure S3. Plot of the absolute residuals of the linear regression.

Table S2. Linear regression parameters and homoscedasticity evaluation.

Parameter	Value
Linear regression equation	y =1,140,726 x + 40,952
Coefficient of determination of the linear regression, R^2	0.9930
Spearman's rank correlation coefficient for homoscedasticity assessment	0.5667
P-value	0.0603
Significance level	0.05
Is homoscedasticity observed?	Yes

Table S3. Accuracy of quantification of the major nusinersen-related impurities by the standard additions method based on XIC chromatograms.

Impurity	Measured content, C_{fact}	Expected content, C_{theor}	Difference, C_{fact} - C_{theor}	Recovery, %
$n-2$	1.02	1.01	0.01	101
	3.02	3.01	0.01	100
	5.05	5.01	0.04	101
	7.08	7.01	0.07	101
	10.01	10.01	0.00	100
$n+1$	2.08	2.02	0.06	103
	4.34	4.02	0.32	108
	6.43	6.02	0.41	107
	8.51	8.02	0.48	106
	11.36	11.02	0.33	103
$n-1$	2.80	2.90	0.10	97
	5.01	4.90	0.11	102
	7.53	6.90	0.63	109
	9.75	8.90	0.85	110
	12.57	11.90	0.67	106
P=O	1.89	1.80	0.09	105
	4.17	3.80	0.37	110
	6.53	5.80	0.73	113
	8.52	7.80	0.72	109
	11.42	10.80	0.62	106

Table S4. Validation: repeatability results (relative XIC peak area).

Component	Replicate						Mean	SD	%RSD
	1	2	3	4	5	6			
P=O	1.23%	1.24%	1.23%	1.25%	1.24%	1.24%	1.24%	0.000	0.79%
Total impurity content ($n-1$)	1.24%	1.28%	1.27%	1.28%	1.28%	1.28%	1.27%	0.000	1.08%
Total impurity content ($n+1$)	0.70%	0.70%	0.69%	0.69%	0.69%	0.68%	0.69%	0.000	0.84%
CNET impurity	<0.02%	<0.02%	<0.02%	<0.02%	<0.02%	<0.02%	<0.02%	-	-
ADP impurity	<0.02%	<0.02%	<0.02%	<0.02%	<0.02%	<0.02%	<0.02%	-	-
IDP impurity	0.38%	0.38%	0.38%	0.38%	0.37%	0.37%	0.38%	0.000	1.17%
2′-O-Me impurity	1.27%	1.28%	1.27%	1.28%	1.27%	1.28%	1.28%	0.000	0.58%
Loss A impurity	0.36%	0.36%	0.36%	0.36%	0.36%	0.36%	0.36%	0.000	0.66%
MAM impurity	<0.02%	<0.02%	<0.02%	<0.02%	<0.02%	<0.02%	<0.02%	-	-
Dithioate impurity	0.59%	0.57%	0.58%	0.58%	0.58%	0.58%	0.58%	0.000	1.00%
Unspecified identified impurities (total)	0.42%	0.40%	0.40%	0.39%	0.42%	0.39%	0.40%	0.000	3.43%
Unspecified unidentified impurities (total)	1.59%	1.69%	1.65%	1.68%	1.59%	1.61%	1.63%	0.000	2.83%
→7354	0.02%	0.02%	0.02%	0.02%	0.02%	0.02%	0.024%	0.001	5.5%
→7015	0.08%	0.09%	0.09%	0.09%	0.08%	0.08%	0.085%	0.002	2.7%
→7337	0.05%	0.07%	0.06%	0.07%	0.06%	0.06%	0.062%	0.005	7.3%
→7090	0.24%	0.25%	0.25%	0.26%	0.25%	0.25%	0.3%	0.010	2.7%
→8275	0.05%	0.04%	0.04%	0.04%	0.04%	0.04%	0.045%	0.002	5.3%
→7046	0.04%	0.05%	0.05%	0.05%	0.05%	0.05%	0.047%	0.003	5.5%
Nusinersen (oligonucleotide purity)	92.23%	92.11%	92.17%	92.10%	92.20%	92.19%	92.17%	0.001	0.06%

Table S5. Validation: intermediate precision results (relative XIC peak area).

Component	Analyst 1						Analyst 2						Mean	SD	%RSD
	1	2	3	4	5	6	1	2	3	4	5	6			
P=O	1.23%	1.24%	1.23%	1.25%	1.24%	1.24%	1.20%	1.20%	1.20%	1.20%	1.20%	1.20%	1.22%	0.000	1.79%
Total impurity content ($n-1$)	1.24%	1.28%	1.27%	1.28%	1.28%	1.28%	1.20%	1.20%	1.20%	1.20%	1.20%	1.20%	1.23%	0.000	3.04%
Total impurity content ($n+1$)	0.70%	0.70%	0.69%	0.69%	0.69%	0.68%	0.73%	0.74%	0.73%	0.73%	0.73%	0.71%	0.71%	0.000	2.75%
CNET impurity	<0.02%	<0.02%	<0.02%	<0.02%	<0.02%	<0.02%	<0.02%	<0.02%	<0.02%	<0.02%	<0.02%	<0.02%	<0.02%	-	-
ADP impurity	<0.02%	<0.02%	<0.02%	<0.02%	<0.02%	<0.02%	<0.02%	<0.02%	<0.02%	<0.02%	<0.02%	<0.02%	<0.02%	-	-
IDP impurity	0.38%	0.38%	0.38%	0.38%	0.37%	0.37%	0.26%	0.34%	0.37%	0.34%	0.25%	0.36%	0.35%	0.000	12.97%
2′-O-Me impurity	1.27%	1.28%	1.27%	1.28%	1.27%	1.28%	1.27%	1.27%	1.27%	1.27%	1.27%	1.28%	1.27%	0.000	0.51%
Loss A impurity	0.36%	0.36%	0.36%	0.36%	0.36%	0.36%	0.37%	0.36%	0.35%	0.34%	0.35%	0.35%	0.36%	0.000	2.12%
MAM impurity	<0.02%	<0.02%	<0.02%	<0.02%	<0.02%	<0.02%	<0.02%	<0.02%	<0.02%	<0.02%	<0.02%	<0.02%	<0.02%	-	-
Dithioate impurity	0.59%	0.57%	0.58%	0.58%	0.58%	0.58%	0.57%	0.58%	0.58%	0.58%	0.58%	0.57%	0.58%	0.000	0.98%
Unspecified identified impurities (total)	0.42%	0.40%	0.40%	0.39%	0.42%	0.39%	0.40%	0.50%	0.50%	0.49%	0.41%	0.50%	0.44%	0.000	11.03%
Unspecified unidentified impurities (total)	1.59%	1.69%	1.65%	1.68%	1.59%	1.61%	1.64%	1.76%	1.76%	1.75%	1.75%	1.75%	1.69%	0.001	4.1%
→*7354*	0.02%	0.02%	0.02%	0.02%	0.02%	0.02%	0.02%	0.02%	0.02%	0.02%	0.02%	0.02%	0.02%	0.000	<0.01%
→*7015*	0.08%	0.08%	0.08%	0.08%	0.08%	0.08%	0.08%	0.09%	0.09%	0.09%	0.08%	0.08%	0.08%	0.000	5.47%
→*7337*	0.05%	0.07%	0.06%	0.07%	0.06%	0.06%	0.05%	0.07%	0.06%	0.07%	0.06%	0.06%	0.06%	0.000	11.61%
→*7090*	0.24%	0.25%	0.25%	0.26%	0.25%	0.25%	0.24%	0.25%	0.25%	0.26%	0.25%	0.25%	0.25%	0.000	2.41%
→*8275*	0.05%	0.04%	0.04%	0.04%	0.04%	0.04%	0.05%	0.04%	0.04%	0.04%	0.04%	0.04%	0.04%	0.000	9.31%
→*7046*	0.04%	0.05%	0.05%	0.05%	0.05%	0.05%	0.04%	0.05%	0.05%	0.05%	0.05%	0.05%	0.05%	0.000	8.08%
Nusinersen (oligonucleotide purity)	92.23%	92.11%	92.17%	92.10%	92.20%	92.19%	92.39%	92.08%	92.08%	92.14%	92.30%	92.13%	92.18%	0.001	0.10%

Table S6. Results of robustness testing during mobile phases aging over 4 days (relative XIC peak area).

Component	Day 1 (rep. 1)	Day 1 (rep. 2)	Day 1 (rep. 3)	Day 2 (rep. 1)	Day 2 (rep. 2)	Day 2 (rep. 3)	Day 3 (rep. 1)	Day 3 (rep. 2)	Day 3 (rep. 3)	Day 4 (rep. 1)	Day 4 (rep. 2)	Day 4 (rep. 3)	RSD, %
Nusinersen, %	93.36	93.42	93.37	93.28	93.29	93.37	93.32	93.39	93.31	93.54	93.58	93.59	0.1
P=O, %	1.11	1.11	1.09	1.32	1.31	1.33	1.23	1.22	1.25	1.10	1.10	1.10	8.1
n-1, %	1.72	1.71	1.70	1.63	1.65	1.62	1.69	1.67	1.69	1.65	1.66	1.61	2.2
n+1, %	0.79	0.76	0.78	0.77	0.76	0.75	0.78	0.77	0.78	0.77	0.77	0.78	1.3
Loss A, Abasic, %	0.43	0.42	0.44	0.41	0.42	0.41	0.42	0.41	0.42	0.41	0.40	0.41	2.3
CNET, %	0.03	0.03	0.02	0.02	0.03	0.03	0.03	0.03	0.03	0.03	-	0.02	15.1
ADP, %	<0.02	<0.02	<0.02	<0.02	<0.02	<0.02	<0.02	<0.02	<0.02	<0.02	<0.02	<0.02	-
IDP, %	0.13	0.13	0.13	0.13	0.14	0.14	0.13	0.13	0.13	0.14	0.12	0.13	2.9
2′-O-CH3, %	0.96	0.94	0.96	0.92	0.91	0.90	0.95	0.93	0.94	0.94	0.93	0.92	1.9
MAM, %	<0.02	<0.02	<0.02	<0.02	<0.02	<0.02	<0.02	<0.02	<0.02	<0.02	<0.02	<0.02	-
2′-O-2-ethoxyethyl / dithioate impurities, %	0.79	0.76	0.79	0.78	0.78	0.76	0.75	0.73	0.74	0.74	0.75	0.75	2.7
Identified unspecified oligonucleotide impurities, %	0.27	0.26	0.27	0.29	0.26	0.27	0.28	0.27	0.27	0.27	0.26	0.28	2.6
Unidentified unspecified oligonucleotide impurities, %	0.42	0.44	0.45	0.45	0.45	0.43	0.41	0.45	0.42	0.42	0.41	0.41	3.9
Total oligonucleotide impurities content, %	6.64	6.58	6.63	6.72	6.71	6.64	6.68	6.61	6.69	6.46	6.42	6.41	1.7

Table S7. Results of robustness testing during sample aging over 24 hours (relative XIC peak area).

Название	0 hrs (rep. 1)	0 hrs (rep. 2)	0 hrs (rep. 3)	24 hours (rep. 1)	24 hours (rep. 2)	24 hours (rep. 3)	RSD, %
Nusinersen, %	93.36	93.42	93.37	93.49	93.41	93.44	0.05
P=O, %	1.11	1.11	1.09	1.16	1.17	1.19	3.43
n − 1, %	1.72	1.71	1.70	1.68	1.69	1.64	1.67
n + 1, %	0.79	0.76	0.78	0.74	0.76	0.76	2.08
Loss A, Abasic, %	0.43	0.42	0.44	0.42	0.41	0.41	2.23
CNET, %	0.03	0.03	0.02	0.03	0.03	0.03	10.77
ADP, %	<0.02	<0.02	<0.02	<0.02	<0.02	<0.02	-
IDP, %	0.13	0.13	0.13	0.13	0.13	0.13	2.08
2′-O-CH3, %	0.96	0.94	0.96	0.90	0.92	0.92	2.43
MAM, %	<0.02	<0.02	<0.02	<0.02	<0.02	<0.02	-
2′-O-2-ethoxyethyl / dithioate impurities, %	0.79	0.76	0.79	0.77	0.76	0.79	1.96
Identified unspecified oligonucleotide impurities, %	0.27	0.26	0.27	0.27	0.27	0.28	2.17
Unidentified unspecified oligonucleotide impurities, %	0.42	0.44	0.45	0.42	0.45	0.40	5.15
Total oligonucleotide impurities content, %	6.64	6.58	6.63	6.51	6.59	6.56	0.75
